# Effects of dietary camelina, flaxseed, and canola oil supplementation on transepidermal water loss, skin and coat health parameters, and plasma prostaglandin E_2_, glycosaminoglycan, and nitric oxide concentrations in healthy adult horses

**DOI:** 10.1093/jas/skad373

**Published:** 2023-11-04

**Authors:** Taylor Richards, Scarlett Burron, Terence Connor McCorkell, Luciano Trevizan, Keely Patterson, Debbie Minikhiem, David W L Ma, Wendy Pearson, Anna K Shoveller

**Affiliations:** Department of Animal Biosciences, University of Guelph, Guelph, Ontario, CanadaN1G 2W1; Department of Animal Biosciences, University of Guelph, Guelph, Ontario, CanadaN1G 2W1; Department of Animal Biosciences, University of Guelph, Guelph, Ontario, CanadaN1G 2W1; Universidade Federal do Rio Grande do Sul, Department of Animal Science, Agronomia, Porto Alegre, RS, Brazil; Department of Animal Biosciences, University of Guelph, Guelph, Ontario, CanadaN1G 2W1; 2306 Skilman Way, Spring Hill, TN 37174, USA; Department of Human Health and Nutritional Sciences, University of Guelph, Guelph, Ontario, CanadaN1G 2W1; Department of Animal Biosciences, University of Guelph, Guelph, Ontario, CanadaN1G 2W1; Department of Animal Biosciences, University of Guelph, Guelph, Ontario, CanadaN1G 2W1

**Keywords:** equine, oil, fatty acids, omega-3, omega-6

## Abstract

Camelina oil is derived from a low-input, high-yield crop and, in comparison to many other dietary fat sources currently used in equine diets, provides a greater amount of α-linolenic acid [ALA; (n-3)], than linoleic acid [LA; (n-6)]. However, no research exists assessing the effects of feeding camelina oil to horses in contrast to other commonly used oils. The objective of this study was to compare the effect of supplementing camelina oil to that of flaxseed and canola oil supplementation, on outcomes related to skin and coat health in horses. Thirty adult horses [23 mares, 7 geldings; 14.9 years ± 5.3 years; 544 ± 66 kg body weight (BW) (mean ± SD)] underwent a 4-week wash-in period consuming hay and sunflower oil. Following the wash-in period, horses were blocked by location, age, and BW, and assigned to one of three treatment oils for 16 weeks (370 mg oil/kg BW): camelina (CAM), canola (OLA), or flaxseed (FLX) oil. Blood samples were collected and plasma prostaglandin E_2_ (PGE_2_; ELISA), nitric oxide (NO; Griess Reaction), and glycosaminoglycan (GAG; DMMB) concentrations were measured on weeks 0 (*n* = 30), 14 (*n* = 24), and 16 (*n* = 30). On weeks 0, 2, 4, 8, and 16, transepidermal water loss (TEWL) was measured pre- and post-acetone application using a VapoMeter (*n* = 26), and a 5-point-Likert scale was used to assess skin and coat characteristics on the side and rump of the horses (*n* = 30). All data were analyzed with repeated measures ANOVA using PROC GLIMMIX in SAS. Independent of treatment, coat color, and quality increased from baseline. There were no differences in the outcomes assessed between the horses supplemented camelina oil and those supplemented canola or flaxseed oil. These results suggest that independent of treatment, all oil supplements improved coat color and quality in horses. This provides indication that camelina oil is comparable to existing plant-based oil supplements in supporting skin and coat health and inflammation in horses.

## Teaser

Camelina oil can provide a new economical and environmentally sustainable source of omega-3 fatty acids that is comparable to flaxseed and canola oil in terms of supporting skin and coat health and inflammation in equine diets.

## Introduction

Dietary fat provides ~2.25 times more energy than an equal weight of digested carbohydrates (Atwater et al., 1990). Following a 10-month acclimation period (consuming a diet with 12% oil inclusion and ~19% total dietary lipid inclusion) horses can tolerate up to 20% oil supplementation in the diet, with a total lipid inclusion of ~27% as digestible energy (DE; Harris et al., 1999). Despite this, standard equine diets consisting of forage, or forage supplemented with concentrates, provide relatively low amounts of fat (2% to 4%; NRC, 2007). Oils are energy-dense dietary fat sources commonly included in equine diets to increase energy content and subsequently reduce reliance on grains to meet energy demands. Supplementing oil in the diet can have numerous physiological benefits for horses, including decreasing thermal load and enhancing metabolic adaptations, both of which have the potential to improve athletic performance (Potter et al., 1990). One of the most notable benefits of including dietary fat sources, like oils, is the provision of essential fatty acids (EFAs; Goh et al., 2004; Harris et al., 1999).

Horses cannot produce the cis-polyunsaturated fatty acids (PUFAs) linoleic acid (LA; C18:2, n-6) and α-linolenic acid (ALA, C18:3; n-3) endogenously, and as a result they must be obtained from the diet. While these fatty acids (FAs), along with their derivatives, help maintain cell membrane integrity, along with neural and retinal development, they also help modulate immunity and inflammation and can support skin and coat health (Goh et al., 2004; Harris et al., 1999; Bazan et al., 2011). The n-6 and n-3 PUFAs compete for the same Δ5- and Δ6-desaturase and elongase enzymes required to convert LA and ALA into their respective long-chain FAs (Hansen et al., 2002). Due to this competitive relationship, an excess of n-6 FAs in the diet can limit the metabolism of n-3 FAs, which can disrupt the homeostasis of the eicosanoids produced from both cascades (Sinnhuber, 1969). The eicosanoids produced from the n-6-derived arachidonic acid (AA; C20:4, n-6) have a different physiological effect than the eicosanoids produced from n-3-derived eicosapentaenoic acid (EPA) and docosahexaenoic acid (DHA). More AA results in pro-inflammatory effects, whereas more EPA and DHA result in resolvins, which are anti-inflammatory and pro-resolving (Calder, 2010). Therefore, dietary supplementation of dietary n-3 FAs may help to suppress inflammatory and allergic reactions and improve the blood lipid profile (Shahidi and Ambigaipalan, 2018). However, the inflammatory eicosanoids produced in the n-6 pathway play an important role in numerous physiological processes, including control of blood flow, vessel dilation, and regulating immunity (Patterson et al., 2012). Thus, sources of both n-3 and n-6 FAs are required to allow sufficient conversion to longer chain FAs in both pathways and avoid the disruption of homeostasis of these physiological processes.

Many oil supplements commonly used in equine diets have a higher concentration of n-6 FAs than n-3 FAs. Common oils and their respective n-6:n-3 ratios include: canola (OLA), 1:0.59; corn, 1:0.01; soybean, 1:0.12; and sunflower, 1:0 (Sarker et al., 2013; Association of American Feed Control Officials, 2020). Flaxseed oil (FLX) and fish oil are frequently used to increase n-3 inclusion; however, fish oil is not an environmentally sustainable choice for meeting the long-term n-3 demands of the growing human population, much less the growing companion animal populations (Sarker et al., 2013). Additionally, fish oil is often not palatable to horses (Jerina et al., 2021). Furthermore, the high levels of ALA in FLX result in poor shelf-life stability and FLX crops are vulnerable to certain climates, seasonal changes, and pests (Muir and Westcott 2003; Sarker et al., 2013). As a result, there is room in the market for an alternative oil supplement that is environmentally and economically sustainable and can provide a rich source of n-3 FAs to contribute to a balanced ratio. In addition to this, there is currently competition for resources in order to meet the demands of both the growing human and animal populations, and as a result, finding ingredient alternatives to sustain both of these food chains and reduce food security risk is highly needed.

*Camelina sativa* is a low-input, high-yield oilseed crop, with a short growing season and performs well in various seasons, climates, and soil types (Putnam et al., 1993; Moser 2010; Vollmann et al., 2015; Berti et al., 2016). The two main derivatives of this crop are meal (~60%) and oil (~40%) (Zubr and Matthäus, 2002). The oil content of Camelina seed falls between 300 and 490 g•kg^−1^ (Zubr 1997; Zubr and Matthäus, 2002; Mupondwa et al., 2016). Camelina oil (CAM) has a desirable n-6:n-3 ratio of 1:1.8, making it a rich source of n-3 FAs (Zubr and Matthäus, 2002). This oil contains naturally high levels of tocopherols and polyphenols, which have been associated with improved skin and coat health and shelf-life stability (Seppanen et al., 2010; Costa et al., 2021). Furthermore, CAM has slightly lower levels of n-3 FAs than FLX making it less susceptible to oxidative rancidity, giving it a better shelf-life by comparison [ALA concentrations (%) for CAM = 28.6-36.7, FLX = 51.0-58.3, and OLA = 9.34-10] (Table 1; Eidhin et al., 2003).

**Table 1.** Fatty acid profile of camelina oil, canola oil, flaxseed oil, and sunflower oil fed at 370 mg oil/kg bodyweight to 30 healthy adult horses enrolled in a skin and coat health trial.

Parameter	Sunflower ^1^	Canola ^2^	Flax ^2^	Camelina ^2^
Saturated Fatty Acids (%)	9.61	6.50	8.20	9.50
Monounsaturated Fatty Acids (%)	14.1	63.8	16.6	35.2
Polyunsaturated Fatty Acids (%)	76.3	29.7	75.2	55.3
Omega 6 (%)	76.2	18.6	16.5	19.8
Omega 3 (%)	0.04	11.1	58.6	35.4
Trans fat (%)	N/A^2^	< 0.1	< 0.1	< 0.1
Total Fat (%)	N/A^2^	99.9	100	99.9

^1^Numerical values are adapted from Kostik et al. (2013) and only represent generic sunflower oil and not the specific brand used for this study (Kostik et al., 2013).

^2^Values equal to means (analyzed in duplicate by SGS Canada Inc.).

Abbreviation: N/A, not available.

Table from Burron et al. (2021).

Supplementing diets with oil supports skin and coat health in numerous species (O’Neill et al., 2002; Neukam et al., 2011; Combarros et al., 2020). However, despite the environmental and storage benefits of camelina oil as opposed to other oils frequently used by the equine industry, to the authors’ knowledge, there are no data directly comparing the effects of CAM supplementation to the effects of other oils being used in equine diets to support skin and coat health and inflammation. Therefore, the objective of this study was to compare the effects of dietary CAM supplementation with those of FLX and OLA on skin and coat health and inflammation in adult horses. Outcomes include: changes in blood biomarkers (prostaglandin E_2_ (PGE_2_), nitric oxide (NO), glycosaminoglycan (GAG)), and skin and coat health parameters. Additionally, skin barrier function and integrity were assessed by measuring transepidermal water loss (TEWL). We hypothesize that CAM (n-3:n-6 = 1.18) is comparable to FLX (n-3:n-6 = 1:4.19) and OLA (n-3:n-6 = 1:0.59) with respect to its effects on plasma PGE_2_, NO, and GAG concentrations, skin and coat health parameters, and TEWL.

## Materials and Methods

### Animals and housing

All experimental procedures were approved by the University of Guelph’s Animal Care Committee (Animal Use Protocol #4481) and were carried out in accordance with national and institutional guidelines for the care and use of animals. Thirty adult horses [*n* = 23, mares; *n* = 7, geldings; 14.9 years ± 5.3 years; 544 ± 66 kg body weight (BW); 6.43 ± 0.992 body condition score (BCS) (Mean ± SD)] were enrolled in this study. Enrolled horses were deemed clinically healthy upon assessment, were not receiving anti-inflammatory medications, and had no abnormalities in routine blood biochemistry and hematology. Three herds of horses from three separate locations were used to complete this study: The Ontario Ministry of Agriculture, Food and Rural Affairs Arkell Research Station (Arkell, ON, Canada; 17 mares, 4 geldings; Horses and research facility owned by the University of Guelph), CJ Equestrian Centre (Rockwood, ON, Canada; 3 mares, 3 geldings; Private facility with privately owned horses), and the University of Guelph Equine Sports Medicine and Reproduction Centre (ESMRC) (Guelph, ON, Canada; 3 mares; Horses and research facility owned by the University of Guelph). All locations are within 20 km of the city of Guelph, Ontario, Canada. All horses were housed outdoors in mixed herds of up to 9 horses and had access to shelters. Due to availability of horses, study start dates were staggered for each location. However, all treatments were equally dispersed across all locations and start dates. The study periods for the Arkell Research, CJ Equestrian, and ESMRC, were June to December, July to December, and August to January, respectively.

### Study design and dietary treatments

Horses housed at all three locations had ad libitum access to hay, although there were minor variations in management techniques between these locations that should be considered. From June to the beginning of October, the horses at the Arkell Research Station were on pasture; thereafter, hay supplemented pasture intake until mid-November, after which time horses were re-located to winter housing (with constant outdoor access) and provided ad libitum access to hay for the remainder of the study period (Supplementary Tables S1 and S2). The ESMRC horses were provided ad libitum access to hay, along with intermittent access to pasture, while the horses at CJ Equestrian had sporadic access to electrolytes, in addition to intermittent access to pasture and ad libitum hay with 1-inch slow feed netting. Neither ESMRC nor CJ Equestrian horses were moved to winter housing during the study period. Prior to enrollment, horses were placed on a 4-week wash-in diet where they were fed sunflower oil at an inclusion rate that was gradually increased from 50 mg oil/kg BW to the final inclusion level of 370 mg oil/kg BW (Table 1). A wash-in period was necessary as it allowed the horses to become gradually acclimated to a diet with high fat-inclusion. This is important since horses lack a gall bladder and must be gradually acclimated to high concentrations of fat. Sunflower oil specifically was chosen as it is the opposite of our treatments in terms of n-6 and n-3 profiles (high in n-6 FAs and low in n-3 FAs (1:0)). This ensures that if effects are observed as a result of n-3 inclusion, it is from our treatment oils, rather than the n-3 FAs found in the wash-in diet.

This study was completed using a randomized complete block design (RCBD). Horses were blocked by location, age, and BW, and randomly assigned to one of three treatment oils: FLX, CAM, or OLA. Throughout the 4-week wash-in period and 16-week treatment period, oil was mixed with soaked hay cubes (Premium Timothy Hay Cubes, ON Dehy, Goderich, ON, Canada) to form a mash, and fed once or twice daily (depending on the location and start date), in addition to free-choice forage. During the 16-week treatment period, oil was provided at the same inclusion level of 370 mg oil/kg BW. Oil intake was recorded daily (Burron et al., under review) and if horses refused treatment diets for more than 2 days consecutively, oil was syringe fed to horses using a 60-mL syringe to ensure delivery of treatment oils. Partial refusals of feed during this study were rare and typically feed would be consumed freely by the horse at a later time on the same day when offered again by researchers.

Assuming that horses at maintenance consume between 1.5% and 2% of their BW in dry matter (DM) daily (National Research Council, 2007), we can infer that the horses in our study, with an average BW of 544 kg, would, at the higher end of this range, be consuming an average of 11 kg of DM daily from either pasture or hay. Considering the relatively low fat content in hay (on average: 2.8% EE on a dry matter basis) and pasture (average ontario pasture: 3.8% EE (DM basis); MadBarn FeedBank), we can estimate that the horses in our study were likely consuming no more than 30-40 g EE per day on a DM basis in their basal diet of hay, pasture, or a combination of both. Due to the group housing of the horses along with their free access to forage, which are both common practices in equine nutrition and welfare, monitoring of forage intake was not feasible during this project. However, given the > 200 g/day of oil supplementation provided daily in comparison to the low EE content in the basal diet, which was standardized among treatment groups, it is unlikely that the daily intake of forage and the lipid profile of the forage would impact the key objective of the present study, which is to compare three dietary oil supplements in terms of their effects on skin and coat health.

As part of additional data collected during this study, in order to trigger a localized immune response, horses were injected with keyhole limpet hemocyanin (KLH) (500 µg) and Quil-A adjuvant (1 g) (mixed into a syringe as a cocktail and injected intramuscularly on the left side of the neck) on weeks 10 and 12 to sensitize horses to KLH. These data are not presented in this manuscript.

### Blood collection

Whole blood (5 mL) was sampled via jugular venipuncture and collected into a sodium heparin plasma vacutainer (Becton, Dickinson and Company, Franklin Lakes, NJ, USA). Upon collection, the vacutainer was gently inverted 8 to 10 times to ensure proper mixing of blood with the anticoagulant, then placed on ice until centrifugation. Samples were centrifuged at 3,500 × *g* for 15 minutes at room temperature (Fisherbrand accuSpin Micro 17 Microcentrifuge, Thermo Fisher Scientific, Waltham, MA, USA). After centrifugation, plasma was separated, and aliquots were frozen at −80 °C until later analysis of blood markers. Blood collection was carried out on weeks 0, 14, and 16; however, on week 14, collection only occurred at two of the three barns (week 0: *n* = 30; week 14: *n* = 24; week 16: *n* = 30).

### Blood markers

Plasma samples from weeks 0, 14, and 16 were analyzed for prostaglandin E_2_ (PGE_2_), glycosaminoglycan (GAG) (dimethyl methylene blue), and nitric oxide (NO). PGE_2_ is a potent, pro-inflammatory mediator derived from the n-6 AA, and its concentrations in the plasma were determined using a commercially available enzyme-linked immunosorbent assay (ELISA) kit (DetectX Prostaglandin E_2_ Enzyme Immunoassay; Arbor Assays, Ann Arbor, MI) (Ricciotti and Fitzgerald, 2011). Samples were run in duplicate according to the manufacturer’s instructions. GAG is an important component of the extracellular matrix (ECM) of the skin, and is a marker of connective tissue turnover, while NO is a marker of oxidative stress and plays a key role in the pathogenesis of inflammation (Sharma et al., 2007; Pfisterer et al., 2021). Plasma concentrations of GAG and NO were determined using spectrophotometric assays (samples were run in duplicate using dimethyl methylene blue and Griess Reaction; Molecular Probes, Eugene, OR, respectively) as previously described by MacNicol et al. (2018). Plasma samples for GAG analysis were diluted 1:10. These particular types of assays do not appear to be species specific and have been previously used in equine research (Bertone et al., 2001; de Grauw et al., 2006; MacNicol et al., 2018).

### Skin barrier function

Transepidermal water loss (TEWL) is commonly used to assess skin barrier function and integrity and is defined as the amount of water that passively evaporates through the skin into the external environment as a result of a water vapor pressure gradient on both sides of the skin barrier (Green et al., 2022). In this study, TEWL was measured using a closed chamber VapoMeter SWL-3 (Delfin Technologies Ltd, Kuopio, Finland) (Validated by De Paepe et al., 2005). During measurement, the VapoMeter is pressed against the skin and a sensor within the chamber monitors the change in relative humidity, and the evaporation rate of water from the skin is automatically calculated based on this change in relative humidity. All horses were previously acclimated to use of the VapoMeter, the researchers and the collection room, prior to the first sample day. Measurements of TEWL were taken on weeks 0, 2, 4, 8, and 16. All body sites were clipped using a 10 blade the day before measurements were taken. Measurements were taken according to the manufacturer’s instructions from the skin over the descending pectoral muscle (chest), medial branch of the flexor carpi ulnaris muscle (inner elbow), ascending pectoralis muscle (withers), and middle gluteal muscle (rump) of the horses, before and after the application of acetone. Acetone application was used to challenge the skin barrier and evaluate its integrity (i.e., a greater difference between pre- and post-acetone TEWL values indicates a weaker skin barrier that is more easily penetrated, in this case by an acetone insult. In comparison, a lower difference between pre- and post-acetone TEWL values indicates a stronger skin barrier, more resilient to environmental insult). Ten pre-acetone and 10 post-acetone measurements were taken per body site and the average of the pre-acetone measurements, and the average of the post-acetone measurements were used for analyses. Once the average of the 10 values were calculated for each sample, any of the 10 values above or below the average by double that of the average or more were considered outliers and removed. All measurements were carried out by a single operator and in the same order of body sites. The horses at the Arkell research station had TEWL measurements taken in an enclosed room with a dehumidifier (and space heater, when necessary) to allow more control over temperature and humidity. The two other barns did not have access to an enclosed room for TEWL measurements, therefore, researchers created a vapor barrier using transparent poly drop sheets and tuck tape to seal around a horse stall and continued to use a dehumidifier (and space heater) to reduce temperature and humidity fluctuations. Despite this, as a result of differing locations and the changing of seasons, temperature ranged from 10 to 33 °C while relative humidity ranged between 43% and 65%. The evaporation rate value is calculated in grams of water per square meter per hour.

### Skin and coat characteristics

Two researchers, blinded to treatment, were trained to perform a subjective skin and coat assessment on weeks 0, 2, 4, 8, and 16 using a 5-point Likert scale (provided in Supplementary Material S3). The Likert scale was used to measure shine, softness, hair quality, color intensity, and moisture of the skin and coat on the horse’s rump and side. To increase consistency among horses, all horses were brushed with 10 strokes on the rump and side, using the same soft-bristled dandy brush immediately prior to each assessment.

### Statistical analysis

Data are presented as mean ± SE unless otherwise indicated. All statistical analyses were performed using the PROC GLIMMIX of SAS Studio® software (v.9.4., SAS Institute Inc., Cary, NC, USA). Horse was treated as the experimental unit, treatment oils and TEWL site were fixed effects, week was a repeated measure, and location was a random effect. An ANOVA was performed to assess the effects of treatment on pre- and post-acetone TEWL averages, circulating blood marker concentrations, and skin and coat health parameters. Assumptions of residuals for all data were assessed using the Shapiro-Wilk normality test. Residuals were not uniformly distributed for TEWL data, and as such, data were log-transformed prior to analysis. Least-square means were used to assess differences in means of treatment, week, TEWL site, TEWL values, difference between pre- and post-acetone TEWL values, treatment by site interactions and treatment by week interactions. Significance was declared at a *P* ≤ 0.05. Trends were declared at *P* ≤ 0.10. When fixed effects were significant, means were separated using Tukey–Kramer adjustments.

## Results

### Plasma PGE_2_, GAG, and NO

**Figure 1.**
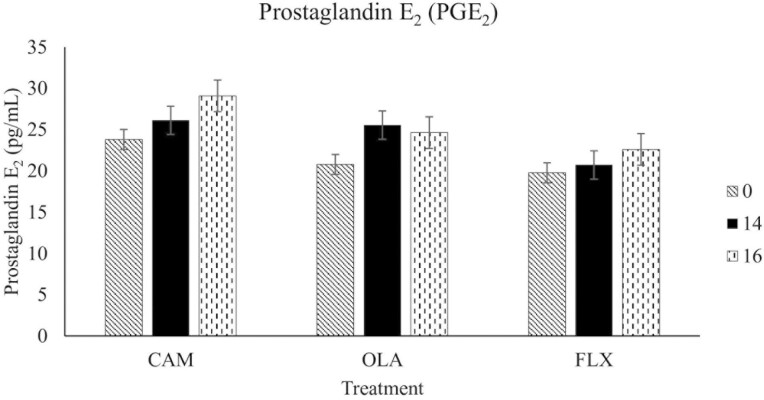
Plasma prostaglandin E_2_ concentrations of 30 healthy adult horses supplemented one of three treatment oils (camelina, canola, flaxseed), and soaked hay cubes, on weeks 0, 14, and 16 of a skin and coat health trial. Treatment oils were provided at an inclusion level of 370 mg oil/kg body weight. CAM, camelina oil; OLA, canola oil; FLX, flaxseed oil.

**Figure 2.**
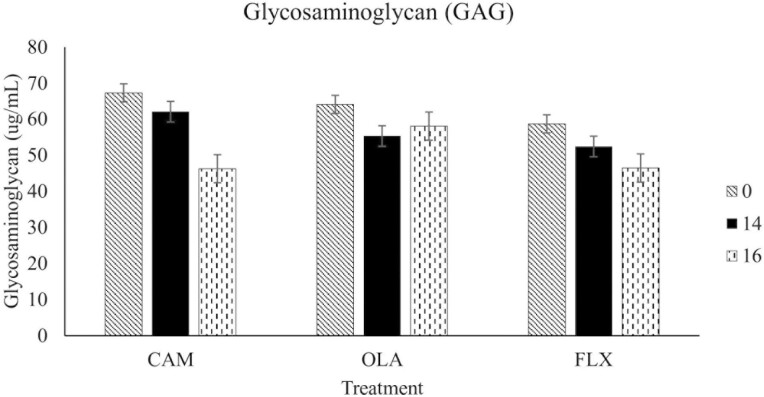
Plasma glycosaminoglycan concentrations of 30 healthy adult horses supplemented one of three treatment oils (camelina, canola, flaxseed), and soaked hay cubes, on weeks 0, 14, and 16 of a skin and coat health trial. Treatment oils were provided at an inclusion level of 370 mg oil/kg body weight. CAM, camelina oil; OLA, canola oil; FLX, flaxseed oil.

**Figure 3.**
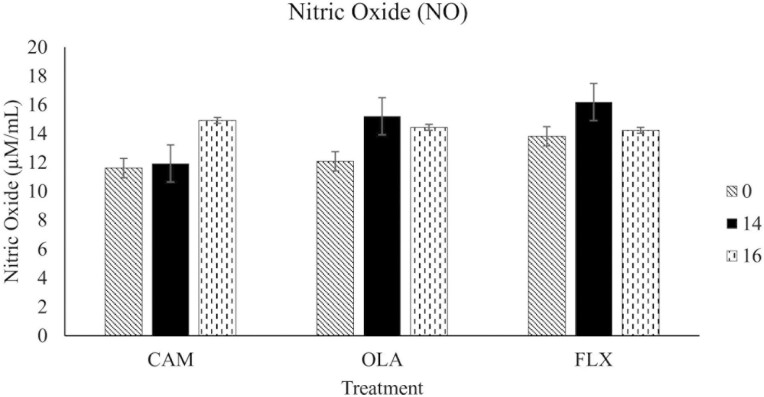
Plasma nitric oxide concentrations of 30 healthy adult horses supplemented one of three treatment oils (camelina, canola, flaxseed), and soaked hay cubes, on weeks 0, 14, and 16 of a skin and coat health trial. Treatment oils were provided at an inclusion level of 370 mg oil/kg body weight. CAM, camelina oil; OLA, canola oil; FLX, flaxseed oil.

The results discussed below are displayed in Figures 1-3.

#### Prostaglandin E_2_

No differences were observed in PGE_2_ concentrations among treatments (*P* = 0.60), between weeks (*P* = 0.74), or for treatment by week interactions (*P* = 0.77).

#### Glycosaminoglycan

Concentrations of GAG were not affected by treatment (*P* = 0.60), week (*P* = 0.39), or treatment by week interaction (*P* = 0.32).

#### Nitric oxide

There were no differences in NO concentrations between treatments (*P* = 0.26), weeks (*P* = 0.16), or treatment by week interactions (*P* = 0.59).

### Transepidermal water loss

The results discussed below are displayed in Table 2.

**Table 2.** Transepidermal water loss (TEWL) (g/m^2^/h) (pre-acetone) presented as LSmeans ± standard error

Treatment	Site	Week					P-values		
		0	2	4	8	16	Treatment	Week*	Site
CAM	Inner elbow	21.03 ± 5.61	19.23 ± 5.61	26.57 ± 5.61	26.42 ± 5.80	14.30 ± 5.61	0.56	0.05	<0.0001
OLA		17.30 ± 5.36	18.71 ± 5.36	39.54 ± 5.36	42.23 ± 5.36	18.37 ± 5.36			
FLX		15.92 ± 5.43	15.03 ± 5.43	36.97 ± 5.43	24.35 ± 5.44	11.77 ± 5.44			
CAM		11.30 ± 5.61	13.41 ± 5.61	28.24 ± 5.61	23.72 ± 5.61	14.33 ± 5.80			
OLA	Chest	11.50 ± 5.36	13.02 ± 5.36	22.50 ± 5.36	31.54 ± 5.36	11.25 ± 5.36			
FLX		11.06 ± 5.43	11.76 ± 5.43	20.39 ± 5.43	22.34 ± 5.44	9.99 ± 5.44			
CAM		14.80 ± 5.61	18.64 ± 5.61	28.25 ± 5.61	28.30 ± 5.61	13.80 ± 5.61			
OLA	Withers	12.42 ± 5.36	19.40 ± 5.36	23.86 ± 5.36	27.72 ± 5.36	18.85 ± 5.36			
FLX		12.22 ± 5.43	13.92 ± 5.43	19.71 ± 5.43	21.21 ± 5.44	10.26 ± 5.44			
CAM		14.60 ± 5.61	12.91 ± 5.61	20.30 ± 5.61	24.70 ± 5.61	13.20 ± 5.61			
OLA	Rump	13.40 ± 5.36	12.53 ± 5.36	16.94 ± 5.36	22.85 ± 5.36	11.13 ± 5.36			
FLX		10.46 ± 5.43	11.30 ± 5.43	19.38 ± 5.43	27.83 ± 5.44	12.21 ± 5.44			

^1^Treatment oils: CAM, camelina; OLA, canola; FLX, flaxseed oil. Oils were provided at an inclusion level of 370 mg oil/kg bodyweight.

*Although the null hypothesis was rejected in the *F*-test (pre-acetone: *P* = 0.05); post-acetone: *P* = 0.04), no significant differences were observed between weeks for pre- and post-acetone TEWL values when pairwise comparisons were analyzed using the Tukey–Kramer adjustment.

*P* ≤ 0.05, repeated measures ANOVA with Tukey post hoc test.

Measurements were taken on the chest, withers, rump, and inner arm of 26 healthy adult horses supplemented one of three treatment oils^1^, and soaked hay cubes, on weeks 0, 2, 4, 8, and 16 of a skin and coat health trial.

Four (#109, #111, #115, and #122) horses were excluded from TEWL measurements as they exhibited signs of stress (i.e., pacing, tail swishing, pawing, sweating) and as such were not successfully acclimated to the enclosed room, leaving a total of 26 horses for TEWL analysis. Of the 10,400 TEWL measurements collected throughout the treatment period, 11 post-acetone values (H = horse(ID#) W = week(week #): H114W0(withers), H118W4(withers), H118W8(inner elbow) (3 values), H118W8(withers) (2 values), H120W4(inner elbow), H120W8(chest) (2 values), H130W0(rump)) were considered outliers and removed.

No differences were observed due to treatment (pre-acetone: *P* = 0.56; post-acetone: *P* = 0.89) or treatment by week interactions (pre-acetone: *P* = 0.80; post-acetone: *P* = 0.13). Additionally, the difference between pre- and post-acetone values, representing barrier recovery, was not impacted by treatment (*P* = 0.58), week (*P* = 0.23), or their interactions (*P* = 0.61). Although the null hypothesis was rejected in the *F*-test (pre-acetone: *P* = 0.05); post-acetone: *P* = 0.04), no significant differences were observed between weeks for pre- and post-acetone TEWL values when pairwise comparisons were analyzed using the Tukey–Kramer adjustment. Furthermore, TEWL averages pre-acetone insult were greater on the inner arm compared to the withers and chest (*P* < 0.0001), and post-acetone values were greater on the inner arm compared to the withers, chest, and rump (*P* = 0.009). Post-acetone TEWL values were greater than pre-acetone TEWL values (*P* < 0.001).

Temperature was split into five categories: A(10°C to 15°C), B(16°C to 20°C), C(21°C to 25°C), D(26°C to 30°C), and E(30°C +). Average TEWL values were greater on sample days that fell under category E in comparison to D, and on sample days that fell under category D compared to A, B, and C (pre-acetone: *P* < 0.001; post-acetone: *P* = 0.004). There was no effect of humidity range on TEWL (pre-acetone: *P* = 0.690; post-acetone: *P* = 0.767).

### Skin and coat health characteristics

**Figure 4.**
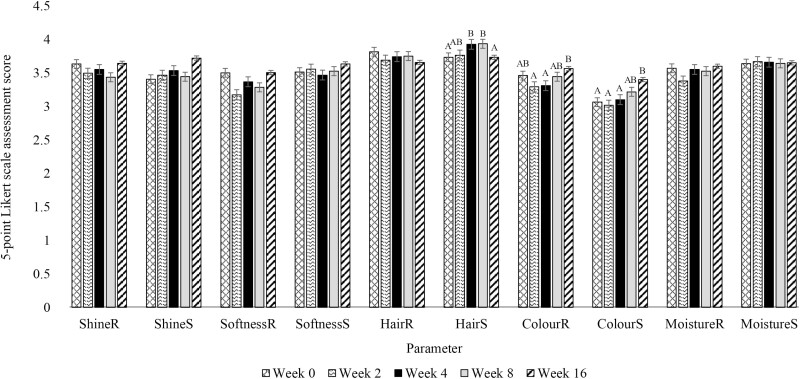
Mean skin and coat health parameter scores of the side (S) and rump (R) assessed using a 5-point Likert scale on 30 adult horses fed one of three treatment oils (Camelina oil, Canola oil, Flaxseed oil) and soaked hay cubes. Treatment oils were provided at an inclusion level of 370 mg oil/kg body weight. ^A, B^ Bars without a common letter differ significantly within each respective parameter (P < 0.05).

The results discussed below are displayed in Figure 4. Assessment sites are denoted by a subscripts: (S) = side; (R) = rump.

#### Shine

There was no effect of treatment (Shine_(S)_: *P* = 0.48; Shine_(R)_: *P* = 0.34), week (Shine_(S)_: *P* = 0.46; Shine_(R)_: *P* = 0.62) or treatment by week interaction (Shine_(S)_: *P* = 0.66; Shine_(R)_: *P* = 0.56) on shine_(S)_ or shine_(R)_.

#### Softness

There was no effect of treatment (Softness_(S)_: *P* = 0.32; Softness_(R)_: *P* = 0.67), week (Softness_(S)_: *P* = 0.78; Softness_(R)_: *P* = 0.38) or treatment by week interaction (Softness_(S)_: *P* = 0.11; Softness_(R)_: *P* = 0.40) on softness_(S)_ or softness_(R)_.

#### Hair quality

Hair_(S)_ quality was lower on weeks 0 compared to weeks 4 and 8, and greater on weeks 4 and 8 compared to week 16 (*P* = 0.0008). There were no differences among treatments (*P* = 0.42), or treatment by week interactions (*P* = 0.43) in hair_(S)_. No differences among treatments (*P* = 0.54), across weeks (*P* = 0.71), or treatment by week interaction (*P* = 0.61) were observed for hair_(R)_.

#### Color

Colour_(S)_ values tended (*P* = 0.09) to be greater with FLX compared to OLA. The CAM treatment was similar in colour_(S)_ compared to FLX or OLA treatments. There was no effect of treatment observed on colour_(R)_ (*P* = 0.13). Regardless of treatment, both color_(R)_ and color_(S)_ were greater on week 16 compared to weeks 2 and 4 (color_(R)_: *P* = 0.02; colour_(S)_: *P* = 0.0009). Additionally, color_(S)_ was greater on week 16 compared to week 0. No differences occurred in color_(R)_ on week 0 or in color_(R)_ and color_(S)_ on week 8. No treatment by week interactions were observed (color_(S)_: *P* = 0.43; color(R): *P* = 0.09).

#### Moisture

There was no effect of treatment (moisture_(S)_: *P* = 0.39; moisture_(R)_: *P* = 0.87), week (moisture_(S)_: *P* = 0.95; moisture_(R)_: *P* = 0.35), or treatment by week interaction (moisture_(S)_: *P* = 0.24; moisture_(R)_: *P* = 0.45) on moisture_(S)_ or moisture_(R)_.

## Discussion

To the authors’ knowledge, this is the first study to investigate the impact of dietary CAM on skin and coat health outcomes in horses in comparison to other commonly used plant-based oils. The results presented herein suggest that CAM is comparable to FLX and OLA in terms of its effects on skin and coat health and can be considered an alternative to these oils.

### Plasma PGE_2_, GAG, and NO

There were no differences in plasma marker concentrations (GAG, PGE_2_, and NO) between the horses supplemented CAM, OLA, and FLX. This is likely a result of the horses all being healthy upon recruitment, with no dermatological issues. Although providing ALA and LA in the diet supports the conversion of these FAs into their respective long chain metabolites via Δ-5 and Δ-6 desaturase and elongase enzymes, the rate of this conversion is low in humans (on average, 1% to 10% of ALA is converted to EPA and 0.5-5% into DHA) and is also believed to be rather limited in dogs (Bibus and Stitt, 1998; Gerster, 1998; Adas et al., 1999; Brenna, 2002; Burdge and Wootton, 2007; Plourde and Cunnane, 2007; Duda et al., 2009; Bauer, 2011; Lenox and Bauer, 2013). Horses that consumed fish oil (a direct source of EPA and DHA) showed marked increases in plasma concentrations of n-3 FAs (including EPA and DHA) compared to the horses fed corn oil (~60% LA) (Hall et al., 2004). This suggests that horses, similar to humans and dogs, may have a limited ability to convert LA and ALA into their long chain PUFAs. However, another study that fed a flaxseed oil enriched pellet for 18 weeks found that horses fed this pellet had greater EPA at all time points (weeks 4, 8, 12, and 16) compared to the non-oil control, suggesting that ALA supplementation increases elongation and desaturation to EPA (Hansen et al, 2002). If our study had enrolled a group of horses supplemented with fish oil, or a non-oil control group to compare, differences in blood markers between treatments may have been observed. However, the goal of this study was to compare camelina oil to existing plant oils with similar compositions. Therefore, it is unlikely that we would observe differences between treatments since the FA concentrations of tissues and plasma are reflective of the composition of oils ingested in monogastric animals. Providing a mixture of marine oils, as a source of EPA and DHA, along with plant oils, such as camelina oil, poses an environmentally and economically sustainable method that reduces the current reliance on marine-based ingredients. However, this is outside of the scope of the present study, which was to compare CAM to OLA and FLX. Further research is warranted to understand the response of horses with subclinical or clinical inflammation fed different plant-based oils or marine-based oils.

### Transepidermal water loss

As expected, TEWL values following barrier disruption via acetone application was greater than TEWL before acetone application. The application of acetone is a common method of barrier disruption, however, to the authors’ knowledge, this is the first study to use this method in horses. Additionally, pre- and post-acetone TEWL values remained similar between treatments and over time in the horses.

One large source of variation throughout this study was the change in temperature and humidity. Although researchers attempted to control such fluctuations on sample days, due to changes in seasonality and lack of an enclosed space at two of the facilities, fluctuations still occurred (31.3 °C in July, 10 °C in December). Additionally, outside of sample days researchers had no control over temperature. Furthermore, not all horses started the study in the same season, resulting in differences in sample times (horses began the wash-in diet in either June, July, or August, with baseline samples being taken 4 weeks later, in either July, August, or September, respectively) and some horses had access to a winter barn mid-way through the study, while others did not. These differences and variability in management are practical and real but make it difficult to determine the actual effect of these oils over time with the current sample size. However, the goal of the present study was not to determine changes due to oil supplementation over time, it was to assess the effects of camelina oil supplementation versus canola and flaxseed oil supplementation in terms of the skin and coat health outcomes measured. Importantly, CAM was comparable to FLX and OLA in terms of its impact on TEWL, barrier integrity, and recovery.

### Skin and coat health assessment

The 5-point Likert scale used in the present study was used to assess parameters including: shine, softness, quality, and color of the coat, along with the moisture of the skin. Color intensity increased from baseline over the 16-week treatment period on both the rump and side of all horses and color intensity on the side tended to be greater in the horses supplemented FLX compared to OLA, while CAM was intermediate. Combarros and colleagues observed that dogs provided with an n-3 capsule [containing 746.5 mg of fish oil (including 230 mg of omega-3, with 160 mg of EPA and 100 mg of DHA, in triglyceride form)] exhibited improved skin and coat characteristics, including reduced coat dullness, with maximal improvements occurring at week 8, in comparison to dogs supplemented a placebo. The researchers attributed these findings to the increased EPA and DHA provided by the supplement (Combarros et al., 2020). The greater color intensity observed in the horses supplemented FLX in the present study may be in part due to the higher concentration of ALA (as compared to OLA) which can be converted to EPA and DHA. Additionally, this may provide an explanation for why the horses consuming CAM had no apparent changes in skin and coat health, as compared to FLX or OLA, as the n-3 content of CAM is intermediate to OLA and FLX. This can be reflected in additional data collected during this project including plasma FAs (not included in this manuscript). This data showed that the ALA concentrations in the horses were significantly greater in the FLX group compared to the OLA group, while CAM was intermediate (Burron et al., under review). However, it is important to note that the dogs enrolled by Combarros and colleagues were considered to have poor quality coats at baseline, and the horses in the present study had relatively good quality coat at baseline and were provided a source of ALA, which is not equally as efficient at providing EPA and DHA as fish oil (Swanson et al., 2012). Similarly, it is important to consider the impact of variables including seasonality, climate, environment, and temperature changes. These management differences and seasonality changes may have created variability in the data, making it difficult to detect differences in the effect of oil supplementation over time (English et al., 2003; Parrado et al., 2019). However, the aim of this study was to determine whether CAM, OLA, and FLX impact skin and coat health differently in healthy horses. Since there were no differences between CAM, OLA, and FLX supplementation, this study suggests that CAM is comparable to FLX and OLA in terms of its effects on skin and coat health characteristics. In addition to TEWL, there is merit in further investigating the effects of season, climate, temperature, and environment on skin and coat health in horses. This will allow researchers to better interpret similar data in the future, as it accurately reflects how a majority of horses are housed and managed. Furthermore, the horses in this study were healthy upon recruitment and not performing significant exercise; future research should explore the impact of supplementing these oils in horses with conditions like atopic dermatitis, and in performance horses doing heavy exercise. Additionally, the amount of oil used in this study is considerably higher than may be used in a barn, and as such, there is merit in conducting dose–response studies with CAM, OLA, and FLX.

## Conclusion

In conclusion, no differences were observed in TEWL, circulating PGE_2_, GAG, and NO, and the skin and coat characteristics measured in this study between the horses receiving CAM, OLA, and FLX. Thus, CAM is comparable to the commonly supplemented oils, FLX and OLA, when considering skin and coat health and inflammatory markers and should be considered as a potential source of n-3 FAs in equine diets, that is, environmentally and economically sustainable. Second, independent of FA provision, all oil supplements improved coat color and quality in healthy horses without inflammation. In order to more accurately assess skin and coat health in horses housed outdoors, more research is required to understand the effects of season, climate, environment, and temperature on TEWL and skin and coat health quality.

## Supplementary Material

skad373_suppl_Supplementary_Tables_S1-S2

## Data Availability

The data presented in this study are available on request from the corresponding author.

## Abbreviations

AA: arachidonic acid
ALA: α-linolenic acid
BW: body weight
CAM: camelina oil
DE: digestible energy
DHA: docosahexanoic acid
DM: dry matter
ECM: extracellular matrix
EFA: essential fatty acid
ELISA: enzyme-linked immunosorbent assay
EPA: eicosapentaenoic acid
ESMRC: Equine Sports Medicine and Reproduction Centre
FA: fatty acid
FLX: flaxseed oil
GAG: glycosaminoglycan
IL-1: interleukin-1
IL-1β: interleukin-1-beta
IL-6: interleukin-6
IL-13: interleukin-13
LA: linoleic acid
MMP: matrix metalloproteinases
NO: nitric oxide
OLA: canola oil
PGE_2_: prostaglandin E_2_
PUFA: polyunsaturated fatty acid
RCBD: Randomized Complete Block Design
TEWL: transepidermal water loss
TIMP: tissue inhibitor of metalloproteinases
TNF-α: tumor necrosis factor alpha
