# Supplementary material for: Effects of dietary camelina, flaxseed, and canola oil supplementation on transepidermal water loss, skin and coat health parameters, and plasma prostaglandin E2, glycosaminoglycan, and nitric oxide concentrations in healthy adult horses
Source: J Anim Sci. 2023 Nov 4;101:skad373. doi: 10.1093/jas/skad373 (PMC10721441; doi:10.1093/jas/skad373)

**Effects of dietary camelina, flaxseed, and canola oil supplementation on transepidermal water loss, skin and coat health parameters, and plasma prostaglandin E_2_, glycosaminoglycan, and nitric oxide concentrations in healthy adult horses**

Taylor Richards^*^, Scarlett Burron^*^, Terence Connor McCorkell^*^, Luciano Trevizan^†^, Keely Patterson^*^, Debbie Minikhiem^#^, David W.L. Ma^||^, Wendy Pearson^*^, and Anna K. Shoveller^*^

*Journal of Animal Science*

(Supplementary Materials S1, S2, S3).

**Supplementary Table S1.** Fatty acid profiles (as-fed) of pasture grass consumed as the basal diet during a 16-week feeding period for horses consuming camelina, flaxseed, or canola oil (370 mg/kg BW/day) at Arkell Research Station.

|  | Pasture^1^ | |
| --- | --- | --- |
|  | June 8^th^ | July 6^th^ |
| Saturated Fatty Acids (%) | 0.400 | 0.520 |
| *cis*-Monounsaturated Fatty Acids (%) | <0.10 | <0.10 |
| *cis*-Polyunsaturated Fatty Acids (%) | 0.250 | 0.320 |
| n-3 Polyunsaturated Fatty Acids (%) | 0.170 | 0.220 |
| n-6 Polyunsaturated Fatty Acids (%) | <0.10 | 0.100 |
| Trans-Fatty Acids (%) | <0.10 | <0.10 |

^1^Pasture samples were collected by mixing 40 small “grab” samples from random locations in the field on the specified date. Samples were submitted to SGS Canada Inc., Agriculture and Feed for analysis. Horses (n=21) at Arkell Research Station, Ontario, Canada were consuming *ad libitum* pasture as the basal diet from June until October, when pasture was additionally supplemented with hay. (Burron et al., under review)

**Supplementary Table S2.** Nutrient analysis of hay^1^ consumed as basal diet during a 16-week feeding period for horses consuming camelina, flaxseed, or canola oil (370 mg/kg BW/day) at Arkell Research Station.

| Test | Dry Matter Basis^2^ | As-Fed Basis^2^ |
| --- | --- | --- |
| Dry Matter (%) | - | 88.58 |
| Moisture (%) | - | 11.42 |
| Protein |  |  |
| Protein % (N x 6.25) | 12.39 | 10.98 |
| SP (%) | 3.61 | 3.20 |
| SP % of CP | 29.14 | - |
| ADF-CP % | 1.72 | 1.52 |
| ADF-CP as % of CP | 13.88 | - |
| NDF-CP% | 6.11 | 5.41 |
| NDF-CP as % of CP | 49.31 | - |
| Fibres |  |  |
| Acid Detergent Fibre (%) | 35.88 | 31.78 |
| aNeutral Detergent Fibre (%) | 58.10 | 51.46 |
| Lignin % | 5.74 | 5.08 |
| Non-Fibres |  |  |
| Ethanol Soluble CHO (%) | 5.56 | 5.81 |
| Water Soluble CHO (%) | 9.31 | 8.25 |
| Non-Structural Carbohydrates | 11.47 | - |
| Starch (%) | 2.16 | 1.91 |
| Fat (%) | 2.04 | 1.81 |
| TFA (% of Fat) | 44.1^3^ | 40.5^3^ |
| 12:0 (%TFA) | 2.83^3^ | 2.60^3^ |
| 14:0 (%TFA) | 1.17^3^ | 1.08^3^ |
| 16:0 (%TFA) | 30.3^3^ | 27.9^3^ |
| 16:1n-7 (%TFA) | 1.42^3^ | 1.31^3^ |
| 18:0 (%TFA) | 3.99^3^ | 3.67^3^ |
| 18:1*trans*-9 (%TFA) | 1.14^3^ | 1.05^3^ |
| 18:1*cis*-9 (%TFA) | 3.32^3^ | 3.06^3^ |
| 18:2n-6 (%TFA) | 18.8^3^ | 17.3^3^ |
| 18:3n-3 (%TFA) | 20.8^3^ | 19.1^3^ |
| Minerals |  |  |
| Ash % | 7.22 | 6.40 |
| Calcium (%) | 0.51 | 0.45 |
| Phosphorus (%) | 0.22 | 0.20 |
| Potassium (%) | 2.46 | 2.18 |
| Magnesium (%) | 0.19 | 0.17 |
| Sodium % | 0.01 | 0.01 |
| Copper (ppm) | 3.77 | 3.34 |
| Iron (ppm) | 69.92 | 61.94 |
| Manganese (ppm) | 39.12 | 34.65 |
| Zinc(ppm) | 23.34 | 20.67 |
| Ca:P Ratio | 2.27 | - |
| Zn:Cu Ratio | 6.19 | - |
| Energy (ADF Based) |  |  |
| TDN (%) | 48.23 | 42.72 |
| Digestible Energy (MCal/Kg) | 2.13 | 1.88 |
| Other |  |  |
| Relative Feed Value | 97.59 | - |

Abbreviations: N = nitrogen; SP = soluble protein; CHO = carbohydrate; TFA = total fatty acids; TDN = total digestible nutrients.

^1^Large bale, 1^st^ cut (2020) hay from Arkell Research Station, Ontario, Canada.

^2^Average values of core samples from three bales in separate areas of storage. Samples were submitted to SGS Canada Inc., Agriculture and Feed for analysis. Horses (n=21) at Arkell Research Station, Ontario, Canada were provided hay *ad libitum* to supplement pasture consumption from October to November 2021, then consumed only hay *ad libitum* from November to December 2021 (end of study).

^3^Values specified represent average fatty acid values from the MadBarn FeedBank for hay with similar %Fat, %NDF, and %CP.

(Burron et al., under review)

**Supplementary Material 3.** A 5-point Likert scale used to assess skin and coat health during a 16-week feeding trial for horses consuming camelina, flaxseed, or canola oil (370 mg/kg BW/day).


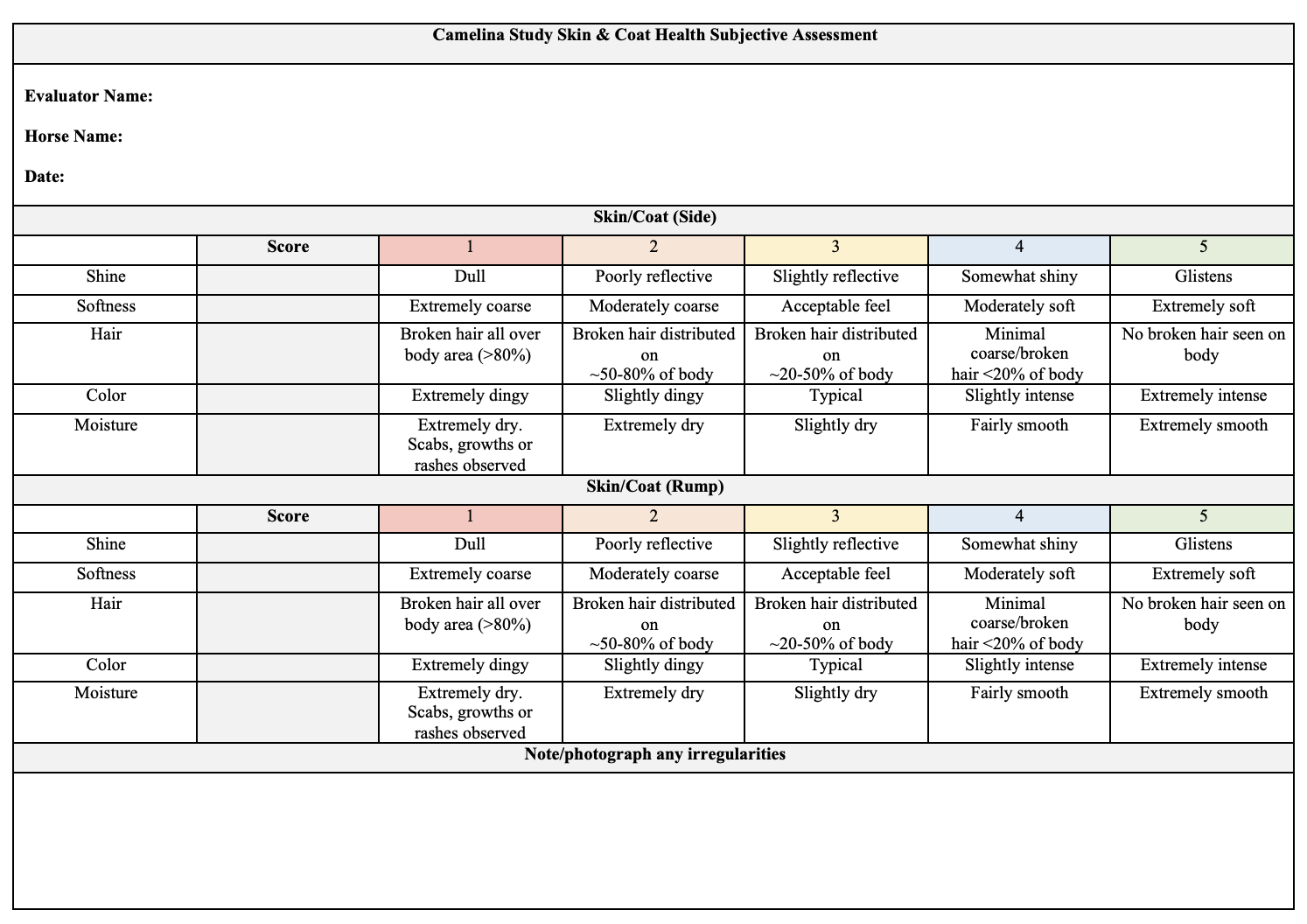

Supplement: skad373_suppl_Supplementary_Tables_S1-S2 [file skad373_suppl_supplementary_tables_s1-s2.docx]
